# Physical Restraint Practices, Attitudes, and Ethical Perceptions Among Intensive Care Unit (ICU) Doctors and Nurses in Morocco: A Cross-Sectional Survey Highlighting the Training-Use Paradox

**DOI:** 10.7759/cureus.110023

**Published:** 2026-06-01

**Authors:** Kaoutar Zirhirhi, Othmane Joutey, Abdelhak Tissir, Sara Lamghari, Lina F Berrada, Sara Chabbar, Fatima Ezzahra Faouji, Anas Mounir, Mohamed Aziz Bouhouri

**Affiliations:** 1 Anesthesiology and Reanimation, Centre Hospitalier Universitaire Ibn Rochd, Casablanca, MAR

**Keywords:** intensive care units, medical ethics, morocco, nursing, patient safety, physical restraint, restraint minimisation

## Abstract

Background: Physical restraint is routinely used in adult intensive care units (ICUs) to prevent patient self-injury and protect medical devices, but is associated with skin injury, prolonged immobilisation, post-traumatic stress, and unresolved ethical tensions. Data from Moroccan ICUs are scarce.

Objectives: This study aimed to describe and compare the practices, attitudes, and ethical perceptions regarding physical restraint among doctors and nurses in adult ICUs of a Moroccan tertiary teaching hospital and to identify factors independently associated with high acceptance of restraint.

Methods: A cross-sectional self-administered questionnaire survey was conducted between June and September 2024 in four adult ICUs of the Ibn Rochd University Hospital complex in Casablanca, Morocco. All doctors and nurses with at least one month of ICU experience were eligible and approached by convenience sampling. The 28-item instrument, developed from a structured literature review and validated by three content experts (pilot n=8), covered demographics, training, practices, attitudes, ethics, legal, and family dimensions. A composite attitude score (range: 4-20) was computed by summing four Likert items (necessity, acceptance, effectiveness, personal experience; Cronbach's α=0.78) and dichotomised at the sample median to define high versus lower acceptance. Analyses used Fisher's exact test, the Mann-Whitney U test, and a multivariable logistic regression limited to three predictors (profession, experience, training) to respect the events-per-variable rule. Reporting follows the Strengthening the Reporting of Observational Studies in Epidemiology (STROBE).

Results: Of 100 eligible staff, 60 returned complete questionnaires (response rate: 60%): 40 doctors (66.7%) and 20 nurses (33.3%). Only 5% of doctors (95% CI: 1.4-16.5) and 15% of nurses (95% CI: 5.2-36.0) had received formal training on restraint. Complications were observed by 72.5% of doctors and 85% of nurses; skin injury was the most prevalent (93.1% and 100%, respectively). Despite this, 77.5% of doctors and 85% of nurses supported maintaining restraint; 72.5% of doctors and 80% of nurses reported restraint use on sedated patients; only 57.5% of doctors and 65% of nurses systematically informed families. In a complete-case analysis (n=51), 56.9% of respondents were classified as high acceptance. In an exploratory multivariable analysis, after adjustment for experience and training, nurses had significantly higher odds of high acceptance than doctors (adjusted OR: 4.89; 95% CI: 1.28-18.7; p=0.021); neither experience nor prior training was independently associated with acceptance.

Conclusion: In this single-centre survey, physical restraint was frequently reported and commonly practised in Moroccan ICUs, with scarce formal training, frequent observed complications, and incomplete family communication: a training-use paradox. Acceptance is shaped more by professional role than by individual exposure or preparation. Mandatory training, standardised protocols, routine sedation and delirium screening, and systematic family engagement are warranted; a multicentre study with patient-level outcomes is the logical next step.

## Introduction

Physical restraint, defined, following the widely used consensus definition of Bleijlevens et al., as any device, material, or equipment attached to or near a person's body that prevents free movement and that the person cannot easily remove [1], is a common practice in adult intensive care units (ICUs) worldwide. Clinicians resort to restraint primarily to prevent the inadvertent removal of endotracheal tubes, central venous catheters, and arterial lines, to manage agitation and delirium, and to reduce the risk of falls in semi-conscious or disoriented patients [2-4]. Despite its ubiquity, restraint is increasingly recognised as a safety intervention with clinically relevant harms and contested ethical foundations.

Observational cohort studies and systematic reviews have consistently associated physical restraint with skin injury, prolonged immobilisation, deep venous thrombosis, nosocomial infection, longer ICU stay, and a higher incidence of post-intensive care syndrome, including post-traumatic stress disorder, among survivors [2,3,5,6]. Concurrently, restraint raises fundamental ethical questions: it limits patient autonomy in settings where patients are frequently unable to provide contemporaneous consent, and its use is often rationalised through a paternalistic appeal to beneficence and non-maleficence [7-10]. International critical care frameworks now advocate restraint minimisation strategies, including protocolised sedation, structured delirium screening, early mobilisation, family presence, and explicit decision pathways, as part of modern intensive care quality [5,11-13].

Data on restraint practices, attitudes, and ethical reasoning among ICU staff in the North African and Maghreb region remain limited. In Morocco specifically, to our knowledge, no national guideline governs restraint use, and no published study has quantified the prevalence of restraint, the extent of staff training, the perception of complications, or the ethical priorities at play in intensive care. Whether the known international tensions, between frequent use, observed harm, and low training, operate similarly in this setting is unknown.

We conducted a cross-sectional survey in four adult ICUs of a large Moroccan teaching hospital. Our primary objective was to assess and compare the practices, attitudes, and ethical perceptions regarding physical restraint between doctors and nurses. A secondary exploratory objective was to identify factors independently associated with high acceptance of restraint in this setting.

## Materials and methods

Study design and setting

We conducted a cross-sectional self-administered questionnaire survey between 1 June 2024 and 30 September 2024 in four adult ICUs of the Ibn Rochd University Hospital complex in Casablanca, Morocco. The participating units comprised one medical ICU and three surgical/polyvalent ICUs, with a combined capacity of 48 beds serving the Greater Casablanca catchment area (≈4.4 million inhabitants). All units operated under the national Ministry of Health framework. At the time of the study, nurse-to-patient ratios ranged from 1:3 to 1:6, no written institutional protocol for physical restraint was in routine use, and structured sedation (Richmond Agitation-Sedation Scale (RASS)) and delirium (Confusion Assessment Method for the Intensive Care Unit (CAM-ICU)) assessments were applied only partially.

Participants and sampling

All doctors (attending physicians and residents) and nurses employed in the four participating ICUs with at least one month of experience working with physical restraint were eligible. Staff rotating through paediatric or gynaecological ICUs were excluded. Participants were recruited by convenience sampling during the study period; staff members present during clinical shifts were approached individually by a member of the research team and invited to participate. Questionnaires were distributed in paper format and completed individually under non-supervised conditions during working shifts. Participants were asked not to discuss their responses with colleagues during questionnaire completion. The number of eligible staff approached, consenting, returning, and analysed is detailed in the Results section. No incentive was offered.

Questionnaire development and validation

A 28-item questionnaire (see Appendices) was developed in four steps. First, a structured narrative review of PubMed, ScienceDirect, and Cochrane (search terms: "physical restraint", "intensive care", "nursing attitudes", "ethics") identified 36 candidate items from six reference studies [7-10,14,15]. Second, the research team adapted these items to the Moroccan context, producing an initial 34-item draft in French. Third, content validity was assessed by three expert reviewers (one intensivist, one senior ICU head nurse, one medical ethicist) who rated each item on a 4-point content validity index (CVI); items with a CVI of <0.75 were revised or discarded (six items removed; four reworded). Fourth, the 28-item instrument was pilot-tested on eight ICU staff from a non-participating unit of the same hospital; pilot respondents were not included in the main analysis. The mean completion time was 12 minutes. The questionnaire was administered in French, the language of medical education in Morocco, and covered four sections: demographics (four items), training and support (three items), practices, perceptions, and attitudes (12 items), and ethical, legal, and family considerations (nine items).

Variables and primary outcome

The primary outcome was high acceptance of physical restraint. It was derived from a composite attitude score computed as the sum of four 5-point Likert items: perceived necessity, acceptance, effectiveness (among those personally involved in restraint application), and personal experience (coded 1=very negative to 5=very positive). These four items were chosen a priori because they map onto a classical attitudinal chain described in health-behaviour research, namely, cognitive appraisal (perceived necessity), evaluative judgement (acceptance), outcome expectancy (perceived effectiveness), and affective/experiential valence (personal experience), each of which contributes a conceptually distinct but coherent facet of an individual's overall stance toward a clinical practice. The composite score ranged from 4 to 20. Internal consistency was acceptable (Cronbach's α=0.78; all item-total corrected correlations >0.45), supporting the operational coherence of the construct. The score was dichotomised at the sample median; tied values at the median were assigned a priori to the high acceptance category. We acknowledge that median dichotomisation reduces statistical power compared with analysis on the continuous score; the approach was chosen a priori for interpretability and for clinical transferability of the high acceptance concept, given the modest sample size and the non-normal distribution of the composite score. Secondary outcomes included ethical justification of restraint, restraint use on sedated patients, observation of complications, and family information. Explanatory variables for multivariable analysis were profession (doctor/nurse), clinical experience (≥5 years/<5 years), and prior formal training on restraint (yes/no).

Statistical analysis

Categorical variables are summarised as counts and percentages with 95% confidence intervals (Wilson score method). Ordinal Likert responses are summarised as medians with interquartile range. Between-profession comparisons used Fisher's exact test for 2×2 tables (preferred over Pearson χ² given the small-sample cell counts), Pearson χ² for multi-category tables with expected counts of ≥5, and the Mann-Whitney U test for ordinal Likert comparisons. Effect sizes are reported as odds ratios with Woolf 95% CI for dichotomous variables, phi or Cramer's V for categorical variables, and rank-biserial correlation for Mann-Whitney. For 2×2 tables containing a sparse cell (n≤1), the OR point estimate is reported, but its magnitude should be interpreted cautiously: statistical significance in such cases is provided by Fisher's exact test, not by the OR confidence interval. Multi-response items were analysed separately by Fisher's exact test per option, with a Benjamini-Hochberg step-up correction for multiple testing (false-discovery rate 5%). A multivariable logistic regression model was fitted with high acceptance as the outcome and profession, experience, and training as predictors; the number of predictors was limited to three to respect an approximate events-per-variable rule of 10 given the modest sample size. Model calibration was assessed by the Hosmer-Lemeshow test and discrimination by Nagelkerke's R². Participants with any missing Likert item were excluded from composite-based analyses (complete-case approach). All tests were two-sided at α=0.05. Given the number of pairwise comparisons performed, results beyond the pre-specified primary outcome should be interpreted as exploratory and hypothesis-generating. Analyses were performed in jamovi 2.3 (open-source, R-based). Reporting follows the Strengthening the Reporting of Observational Studies in Epidemiology (STROBE) [16]. The anonymized dataset and data dictionary are available from the corresponding author upon reasonable request.

Ethical considerations

The protocol was reviewed and approved by the Biomedical Research Ethics Committee of the Faculty of Medicine and Pharmacy of Casablanca, Hassan II University (approval number: CERBC-2024/042; date: 15 May 2024). The study complied with the Declaration of Helsinki (2013 revision) [17] and with Moroccan Law 09-08 on the protection of personal data. All participants received a written information sheet and provided informed written consent before completing the questionnaire; no patient-identifying data were collected. Participation was voluntary, and participants could withdraw at any time without consequences.

## Results

Participants

Of 100 ICU staff approached in the four participating units, 92 were eligible, 85 consented, 66 returned questionnaires, and 60 returned complete questionnaires (response rate: 60%; 95% CI: 50.1-69.2). Six participants were excluded for more than 20% missing items. The analysed cohort comprised 40 doctors (66.7%) and 20 nurses (33.3%). Baseline characteristics by profession are shown in Table 1. The mean age was 27 years in doctors (range: 23-42) and 26 years in nurses (range: 22-41). Male sex predominated among doctors (60%) and female sex among nurses (60%). Most respondents had 1-5 years of ICU experience (57.5% of doctors and 65% of nurses); no doctor and two nurses reported >10 years of experience.

**Table 1:** Baseline characteristics of respondents by profession (N=60) †: Mann-Whitney U test; ‡: Fisher's exact test; §: Pearson χ² (df=3; χ²=7.11; V=0.34)

Characteristic	Doctors (n=40)	Nurses (n=20)	Overall (N=60)	P-value
Age (years), mean (range)	27 (23-42)	26 (22-41)	27 (22-42)	0.620†
Sex, n (%)				
Male	24 (60)	8 (40)	32 (53.3)	0.176‡
Female	16 (40)	12 (60)	28 (46.7)	
Experience, n (%)				
<1 year	13 (32.5)	2 (10)	15 (25)	0.068§
1-5 years	23 (57.5)	13 (65)	36 (60)	
5-10 years	4 (10)	3 (15)	7 (11.7)	
>10 years	0 (0)	2 (10)	2 (3.3)	

Training and support

Formal training on physical restraint was rare in both groups: two of 40 doctors (5%; 95% CI: 1.4-16.5) and three of 20 nurses (15%; 95% CI: 5.2-36.0) had received any specific training; the difference was not statistically significant (Fisher's exact; p=0.322). Among the few trained respondents, the training was rated as only slightly or moderately effective. Conversely, 60% of doctors and 50% of nurses considered additional training on restraint to be extremely beneficial (p=0.586), and none of the respondents rated further training as not at all beneficial, with the exception of one nurse.

Practices and indications

Personal involvement in applying restraint was nearly universal: 37 of 40 doctors (92.5%) and all 20 nurses (100%) reported having been personally involved. Among the four most frequently cited triggers for restraint, three differed significantly between doctors and nurses (Table 2). Doctors were more likely than nurses to invoke risk of violence to self (97.5% vs. 75%; p=0.013), risk of violence to others (82.5% vs. 50%; p=0.014), and lack of alternatives (52.5% vs. 15%; p=0.006); the latter three comparisons remained significant after Benjamini-Hochberg correction (q=0.033). Agitation and risk of fall were cited frequently by both groups without significant difference. Of note, restraint use on sedated patients was reported by 29 of 40 doctors (72.5%) and 16 of 20 nurses (80%), and 77.5% of doctors and 85% of nurses supported maintaining restraint as a clinical tool in the ICU.

**Table 2:** Practices and indications for physical restraint by profession *: odds ratio (Woolf 95% CI); **: wide confidence interval due to sparse cell (one doctor did not cite this trigger; statistical significance is given by Fisher's p-value, not by the OR point estimate); †: remain significant after Benjamini-Hochberg correction (q=0.033 each)

Item	Doctors, n (%)	Nurses, n (%)	OR (95% CI)*	P-value
Personal involvement in applying restraint	37 (92.5)	20 (100)	-	0.544
Restraint used on sedated patients	29 (72.5)	16 (80)	0.66 (0.18-2.45)	0.753
Support maintaining restraint in the intensive care unit	31 (77.5)	17 (85)	0.61 (0.15-2.50)	0.734
Trigger				
Agitation	37 (92.5)	17 (85)	2.18 (0.40-11.8)	0.390
Risk of violence to self	39 (97.5)	15 (75)	13.00 (1.40-117)**	0.013†
Risk of violence to others	33 (82.5)	10 (50)	4.71 (1.45-15.2)	0.014†
Risk of fall	37 (92.5)	15 (75)	4.11 (0.91-18.3)	0.103
Lack of alternatives	21 (52.5)	3 (15)	6.26 (1.59-24.7)	0.006†
Patient non-cooperation	14 (35)	9 (45)	0.66 (0.22-1.94)	0.575
Workload/staff shortage	4 (10)	2 (10)	1.00 (0.17-5.97)	1.000

Attitudes toward restraint

Attitudinal responses on the 5-point Likert items are summarised in Table 3. Doctors and nurses did not differ significantly on perceived necessity (Mann-Whitney U=302.5; p=0.104) or on acceptance (U=361.5; p=0.525). Nurses, however, rated the effectiveness of restraint more favourably than doctors (U=248.0; p=0.032; rank-biserial r=0.33) and reported more positive personal experiences with its use (U=230.0; p=0.037; r=0.32). Both findings suggest a more positive stance toward restraint among nurses at the individual item level.

**Table 3:** Attitudes and complications related to physical restraint by profession MW: Mann-Whitney U; *: p<0.05 Composite score computed on 51 complete cases (nine doctors excluded for missing effectiveness or experience item).

Item	Doctors	Nurses	Test	P-value
Likert medians (IQR)				
Necessity (1-5)	3 (3-4)	4 (3-4)	MW U=302.5	0.104
Acceptance (1-5)	4 (3-4)	4 (3-4)	MW U=361.5	0.525
Effectiveness (1-5)*	3 (3-4)	4 (4-5)	MW U=248.0	0.032
Personal experience (1-5)*	2 (2-3)	4 (2-4)	MW U=230.0	0.037
Composite score (range: 4-20)				
Median (IQR): complete cases	13 (12-14)	15 (14-16)	MW U=180.0	0.004
High acceptance (score ≥14), n (%)	13/31 (41.9)	16/20 (80)	Fisher	0.010
Complications observed (any)				
Any complication observed, n (%)	29/40 (72.5)	17/20 (85)	Fisher	0.347
Skin injuries	27/29 (93.1)	17/17 (100)	Fisher	0.524
Negative psychological effects*	19/29 (65.5)	5/17 (29.4)	Fisher	0.031
Deterioration of mental state	12/29 (41.4)	5/17 (29.4)	Fisher	0.533
Muscle atrophy	9/29 (31)	8/17 (47.1)	Fisher	0.349

Complications and their impact

Complications linked to restraint were observed by the large majority of both professional groups: 72.5% of doctors and 85% of nurses (p=0.347; Table 3). Skin injury, namely, abrasions, erythema, and pressure ulcers, was nearly universal among observers (93.1% of doctors; 100% of nurses). The pattern of other complications differed: doctors reported negative psychological effects (loss of control, frustration) significantly more often than nurses (65.5% vs. 29.4%; p=0.031), while nurses reported more muscle atrophy (47.1% vs. 31%) and similar rates of mental state deterioration and circulatory and respiratory complications. Among those who had observed complications, 65.5% of doctors but only 47.1% of nurses reported that these observations had changed their view on restraint (p=0.352).

Ethical, legal, and family dimensions

A majority in both groups considered physical restraint ethically justified in their medical practice, 29 of 40 doctors (72.5%) and 13 of 20 nurses (65%), without significant difference (p=0.564; Table 4). The ethical principles most frequently prioritised were non-maleficence (60% in both groups) and beneficence (55% in both groups); patient autonomy was selected by only 20% of respondents in each group and justice by only 10%. Legal considerations were reported to enter the decision in 55% of doctors and 60% of nurses (p=0.787), with collective team involvement the most cited legal safeguard (75% of doctors; 85% of nurses). Family information was systematic in 57.5% of doctors and 65% of nurses (p=0.780); when informed, families were rated as broadly accepting of the decision. A family-level emotional impact had been observed by 57.5% of doctors and 40% of nurses (p=0.275).

**Table 4:** Ethical, legal, and family dimensions by profession

Item	Doctors, n/N (%)	Nurses, n/N (%)	OR (95% CI)	P-value
Restraint ethically justified	29/40 (72.5)	13/20 (65)	1.42 (0.43-4.73)	0.564
Ethical principle: non-maleficence	24/40 (60)	12/20 (60)	1.00 (0.33-3.00)	1.000
Ethical principle: beneficence	22/40 (55)	11/20 (55)	1.00 (0.34-2.93)	1.000
Ethical principle: patient autonomy	8/40 (20)	4/20 (20)	1.00 (0.25-4.04)	1.000
Legal aspects considered	22/40 (55)	12/20 (60)	0.81 (0.28-2.40)	0.787
Collective team involvement in decision	30/40 (75)	17/20 (85)	0.53 (0.13-2.19)	0.523
Family systematically informed	23/40 (57.5)	13/20 (65)	0.73 (0.24-2.21)	0.780
Family-level impact observed	23/40 (57.5)	8/20 (40)	2.03 (0.68-6.03)	0.275

Factors associated with high acceptance of restraint (multivariable analysis)

In the analytical cohort of 51 participants with complete Likert data, 29 (56.9%) were classified as high acceptance (composite score ≥14). The proportion was 41.9% (13/31) among doctors and 80% (16/20) among nurses (Fisher's exact p=0.010). Results of univariable and multivariable logistic regression are shown in Table 5. After adjustment for clinical experience and prior training, nurses had significantly higher odds of high acceptance than doctors (adjusted OR: 4.89; 95% CI: 1.28-18.7; p=0.021). Neither clinical experience ≥5 years (aOR: 2.22; 95% CI: 0.38-12.9; p=0.370) nor prior formal training (aOR: 2.15; 95% CI: 0.21-21.9; p=0.520) was independently associated with acceptance. The model had acceptable calibration (Hosmer-Lemeshow χ²=2.87; p=0.720) and modest discrimination (Nagelkerke R²=0.18). Given the small number of events per category, especially for the training variable (only five trained participants), the model should be interpreted as exploratory, underpowered, and hypothesis-generating rather than as a definitive risk prediction tool.

**Table 5:** Univariable and multivariable logistic regression of factors associated with high acceptance of physical restraint (n=51) *: adjusted for the other two predictors Model fit: Hosmer-Lemeshow χ²=2.87; p=0.720. Discrimination: Nagelkerke R²=0.18

Predictor	OR (95% CI)	P-value	aOR (95% CI)*	P-value	n high/n total
Profession: nurse vs. doctor	5.54 (1.50-20.5)	0.010	4.89 (1.28-18.7)	0.021	16/20 vs. 13/31
Experience: ≥5 years vs. <5 years	2.61 (0.47-14.4)	0.270	2.22 (0.38-12.9)	0.370	6/8 vs. 23/43
Training: yes vs. no	3.36 (0.35-32.5)	0.340	2.15 (0.21-21.9)	0.520	4/5 vs. 25/46

**Figure 1:**
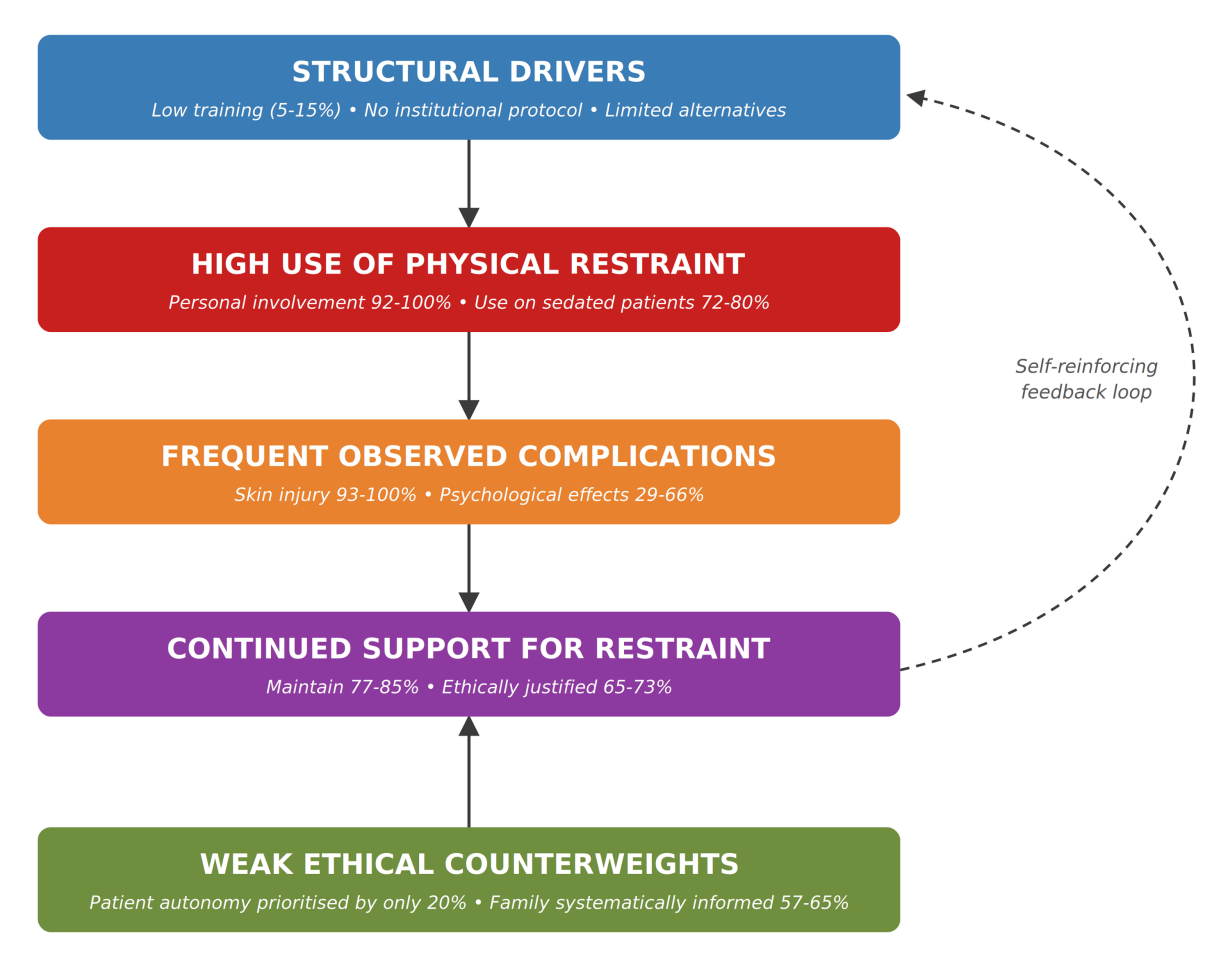
Conceptual framework of the restraint paradox in ICU practice ICU: intensive care unit Five interacting dimensions define the restraint paradox in this setting: structural drivers create high use, which produces frequent observed complications, yet continued support is maintained because the ethical counterweights (autonomy, family communication) carry insufficient weight in practice. Percentages refer to doctors/nurses in the current study (n=60); they represent staff-reported practices and perceptions, not patient-level prevalence data. The figure was created using Microsoft PowerPoint (Microsoft Corporation, Redmond, Washington, United States).

These structural drivers, high use, observed complications, and persistent support, together with the weak ethical counterweights, are summarised in Figure 1.

## Discussion

In this first cross-sectional survey of ICU staff in Casablanca, physical restraint was frequently reported and commonly practised in this setting, its complications were frequently observed, and yet both doctors and nurses broadly supported the continuation of the practice, a training-use paradox sustained despite scarce formal training and low prioritisation of patient autonomy. After adjustment for experience and training, professional role was the only factor independently associated with high acceptance of restraint, nurses being substantially more supportive than doctors, consistent with prior qualitative work documenting more permissive nursing beliefs around restraint use [4]. It is important to emphasise that this apparent paradox is descriptive and hypothesis-generating rather than statistically demonstrated: our data document the co-existence of these four dimensions but do not test a directional causal link between them.

Our findings align, in several dimensions, with the international literature. The prevalence of restraint use on ventilated patients in our cohort (92-100% of staff personally involved) is consistent with rates described in the international literature, where reported proportions of mechanically ventilated ICU patients exposed to restraint range from 50% to 97% across studies [2,6,7,14,18]. The scarcity of formal training in our cohort (5-15%) appears lower than rates reported in European and Anglophone ICUs, where authors describe a broad training gap in the literature [3], suggesting a regional training shortfall. The near-systematic use of restraint on sedated patients (72-80%) is markedly higher than the lower rates reported in some settings, such as in South Africa [15], and may reflect the lower availability of continuous sedation monitoring and of delirium screening tools in our units. The rate of systematic family information (57-65%) lies within the broad range described internationally: some cohorts report low family involvement in restraint decisions, while others describe much higher rates, reflecting wide variability across countries and clinical cultures [7,10,19]. The low prioritisation of patient autonomy (20%) contrasts with the more autonomy-centred reasoning described in international critical care ethics literature [9-11]. These international comparisons should be read with caution: the settings described differ substantially in health system organisation, nurse-to-patient staffing, availability of structured sedation and delirium protocols, access to continuous monitoring, and cultural norms surrounding family involvement in clinical decisions. Our figures therefore reflect not only the attitudes of Moroccan ICU staff but also the organisational and resource context in which those attitudes are formed and should be positioned alongside, rather than directly equated with, published rates from higher-resource or differently structured systems.

The paradox itself (high use, observed harm, and continued support) warrants a structural interpretation rather than an ethical one. Our data suggest that the persistence of restraint appears less shaped by ethical conviction than by structural factors: absence of institutional protocols, limited training, constrained nurse-to-patient ratios (1:3 to 1:6), and the absence of accessible alternatives such as routine delirium screening, protocolised sedation interruption, or dedicated sitter personnel. In this context, restraint may partly operate as a default safety strategy rather than as a deliberated clinical decision, and its ethical justification remains similar across groups (~70%) precisely because the ethical counterweights, such as autonomy, dignity, and transparent family engagement, are underdeveloped in the local practice culture. The observed prioritisation of non-maleficence and beneficence (~60% each) over autonomy (20%) is also coherent with the broader clinical context: ICU patients are frequently unable to participate in real-time decision-making, decisions are often made under time pressure, and the prevailing medical culture in the region has historically leaned toward a more paternalistic model of care. These cultural and contextual elements should be read as facilitators, rather than as justifications, of the current imbalance between ethical principles.

The finding that profession is the only independent predictor of high acceptance, while training and experience are not, is compatible with this interpretation: the observed association between profession and acceptance may reflect differences in bedside exposure, in the proximity of agitated patients, and in the immediate safety imperatives that nurses encounter more frequently than doctors, rather than an intrinsic professional attitude. The absence of association between prior training and acceptance may reflect the extremely small number of trained participants (n=5) and insufficient statistical power to detect a real effect or, alternatively, that the training actually received was too limited in content or duration to alter attitudes; these two explanations cannot be disentangled in our data.

The clinical implications are direct. First, formal training on physical restraint, currently received by only 5-15% of staff, should be made mandatory during residency and nursing induction, with refresher modules integrated into ICU quality programmes. Second, a locally adapted restraint protocol is needed, specifying indications, time-limited orders (e.g., maximum 24 hours with structured reassessment), documentation requirements, and escalation/de-escalation pathways. Third, routine sedation (RASS) and delirium (CAM-ICU) assessments should be operationalised in every unit, consistent with the 2018 Pain, Agitation/Sedation, Delirium, Immobility, and Sleep (PADIS) guidelines [5], to reduce the reliance on restraint as a first-line response to agitation. Fourth, family communication should be systematised and supported by a brief information leaflet; our data suggest that when families are informed, acceptance appears achievable in this context, although this finding rests on staff self-report and may be overestimated. A concise clinical implication box is provided in Table 6.

**Table 6:** Operational implications: three levels of action for restraint minimisation in Moroccan ICUs Three-tier action plan derived from study findings: bedside (ICU team), institutional (hospital administration), and national (Ministry of Health) levels. RASS: Richmond Agitation-Sedation Scale; CAM-ICU: Confusion Assessment Method for the Intensive Care Unit; PADIS: Pain, Agitation/Sedation, Delirium, Immobility, and Sleep; SCCM: Society of Critical Care Medicine; ICU: intensive care unit; PTSD: post-traumatic stress disorder

Level	Stakeholder (timeframe)	Concrete actions
Level 1	ICU team (actionable tomorrow)	• Bedside: document every restraint order with written indication, start time, and planned reassessment interval
		• Integrate RASS and CAM-ICU into routine nursing rounds three times per shift
		• Systematically inform the patient's family at the time of restraint application, using a standardised two-sentence verbal explanation
		• Reassess every restrained patient at each medical round; de-escalate whenever clinically possible
Level 2	Hospital administration (within six months)	• Develop and disseminate an institutional written protocol on physical restraint with time-limited orders (maximum 24 hours, renewable only on medical reassessment), structured reassessment intervals, and documentation requirements
		• Deliver a mandatory two-hour training module on physical restraint to all ICU staff during induction and once annually; track completion as a quality indicator
		• Produce a brief printed information leaflet for families explaining why restraint is used, what it is, and what to expect; distribute at ICU admission
		• Audit restraint-free ICU days as a monthly quality indicator and feed the result back to each unit
Level 3	Ministry/national level (within two years)	• Commission a national consensus on physical restraint in adult ICUs, adapted to the Moroccan context and aligned with PADIS/SCCM recommendations
		• Integrate formal restraint training into the national curricula of medical residency programmes and nursing schools
		• Fund a multicentre Moroccan prevalence study with patient-level outcomes (delirium, pressure ulcers, ICU length of stay, post-ICU PTSD) to establish a national baseline before implementing a restraint minimisation bundle nationwide

Limitations

Our study has several limitations. It is a single-centre survey in one Moroccan university hospital complex, limiting generalisability to other regions or to private sector ICUs. The sample size was modest (n=60; n=51 in the multivariable analysis), which limited statistical power to detect small or moderate between-group differences and led to wide confidence intervals, particularly for items with near-zero cell counts, where odds ratios should be read as descriptive signals rather than as precise effect magnitudes. The point estimate for training in the multivariable model should therefore be read as directional only, given the very small number of exposed participants (5 of 51; 9.8%) who reported any formal training; statistical power to detect a true effect on this variable was severely limited, and the observed non-association should not be interpreted as evidence of no effect. Training was recorded as a binary variable (yes/no) only; no data on duration, format, content, recency, or quality of training were collected, and exposure misclassification cannot be excluded. Convenience sampling may have introduced selection bias toward more engaged or more opinionated staff, and non-response bias (40% non-response) cannot be excluded. All measures were self-reported and were not triangulated against medical records; social desirability may have inflated the reported rates of ethical reasoning, documentation, and family communication, including perceived family acceptance. Several potentially important confounders were not captured and could not be adjusted for, including real-time workload, individual nurse-to-patient ratio at the time of restraint decision, patient severity (SAPS II/SOFA), incident agitation or delirium prevalence, and ICU-level organisational factors; residual confounding is therefore possible. The instrument, although content-validated and pilot-tested with acceptable internal consistency (Cronbach's α=0.78 for the attitudes subscale), did not undergo formal external validation, test-retest reliability assessment, or cross-cultural validation; reproducibility across different institutional or cultural settings therefore remains uncertain, and measurement error cannot be fully quantified.

Future research

Two priorities emerge for future research. A multicentre Moroccan prevalence and practice study, including private sector and peripheral public ICUs, would test generalisability and establish a national baseline. A before-after implementation study of a restraint minimisation bundle [12,13,20-22], protocolised sedation, routine CAM-ICU, mandatory staff training, and structured family engagement, with restraint-free days and patient-level outcomes (incident delirium, skin injury, post-ICU symptoms) as primary endpoints would quantify the clinical benefit of correcting the structural drivers identified here.

## Conclusions

Physical restraint is widely used, its complications are frequently observed, and its continued use is broadly supported by both doctors and nurses in the Moroccan ICUs of this study, a training-use paradox. Only 5-15% of staff have received any formal training, and patient autonomy is the lowest-prioritised ethical principle in both groups. Profession is the only factor independently associated with high acceptance of restraint; neither individual clinical experience nor prior training modifies that association. These findings argue for three concrete institutional actions: mandatory protocolised training, a national intensive care restraint protocol with time-limited orders and routine sedation-and-delirium screening, and systematic family engagement. A multicentre prospective cohort with patient-level outcomes is the logical next step.

## Appendices

Study questionnaire

The following is an English translation of the questionnaire, which was developed by the authors and administered in French to physicians and nurses working in intensive care units in Morocco. Items marked with a checkbox (☐) indicate single- or multi-select options; items with an underscore (___) indicate free-text or numeric responses.

Section 1. Demographic Information

**1. Age (years):** _____ 

2. Sex:

- ☐ Female
- ☐ Male
- ☐ Prefer not to say

3. Profession:

- ☐ Intensive care physician/resident
- ☐ Registered nurse
- ☐ Nursing assistant
- ☐ Other (please specify): _____

**4. Years of professional experience in critical care:** ___ years

Section 2. Training and Support

5. Have you received specific training on the use of physical restraint?

- ☐ Yes
- ☐ No

6. If yes, how would you rate this training?

- ☐ Very poor
- ☐ Poor
- ☐ Acceptable
- ☐ Good
- ☐ Excellent

7. Do you think additional training on this topic would be beneficial for healthcare professionals?

- ☐ Yes
- ☐ No
- ☐ Unsure

Section 3. Perceptions and Attitudes

**8. What factors lead you to consider the use of physical restraint?** *(Select all that apply)*

- ☐ Patient agitation
- ☐ Risk of self-extubation
- ☐ Risk of removing catheters, lines, or medical devices
- ☐ Aggressive behavior
- ☐ Confusion or delirium
- ☐ Risk of falls
- ☐ Insufficient staffing
- ☐ Other (please specify): ___

9. What is your overall attitude toward the use of physical restraint?

- ☐ Strongly unfavorable
- ☐ Unfavorable
- ☐ Neutral
- ☐ Favorable
- ☐ Strongly favorable

10. How do you perceive the acceptance of physical restraint in critical care units by the medical staff?

- ☐ Not accepted at all
- ☐ Rarely accepted
- ☐ Sometimes accepted
- ☐ Often accepted
- ☐ Fully accepted

11. Have you personally participated in the application of physical restraint?

- ☐ Yes
- ☐ No

12. If yes, how would you rate the effectiveness of physical restraint in those situations?

- ☐ Not effective
- ☐ Slightly effective
- ☐ Moderately effective
- ☐ Very effective
- ☐ Highly effective

13. On the following scale, how would you describe these experiences?

- ☐ Very negative
- ☐ Negative
- ☐ Neutral
- ☐ Positive
- ☐ Very positive

14. Have you observed consequences or complications associated with the use of physical restraint?

- ☐ Yes
- ☐ No

**15. If yes, please specify the complications or consequences observed.** *(Select all that apply)*

- ☐ Skin lesions or pressure injuries
- ☐ Worsening of agitation
- ☐ Psychological distress/trauma
- ☐ Impaired circulation in the restrained limb
- ☐ Worsening of delirium
- ☐ Nerve injury
- ☐ Self-injury despite restraint
- ☐ Other (please specify): ___

16. Do you think these complications have influenced your opinion on physical restraint?

- ☐ Yes
- ☐ No
- ☐ Unsure

17. What is your opinion regarding the continued use of physical restraint in intensive care?

- ☐ Should be abandoned
- ☐ Should be significantly reduced
- ☐ Should remain at current levels
- ☐ Should be expanded when needed
- ☐ No opinion

**18. What alternatives to physical restraint do you propose?** *(Select all that apply)*

- ☐ Pharmacological sedation/analgesia adjustment
- ☐ Verbal de-escalation techniques
- ☐ Increased family presence
- ☐ Specialized one-to-one monitoring (sitter)
- ☐ Environmental modifications (lighting, noise reduction)
- ☐ Reorientation and cognitive stimulation
- ☐ Other (please specify): ___

19. Has your unit ever used physical restraint on sedated patients?

- ☐ Yes
- ☐ No
- ☐ Unsure

Section 4. Ethical and Legal Considerations

20. Do you think the use of physical restraint is ethically justified in medical practice?

- ☐ Never justified
- ☐ Rarely justified
- ☐ Sometimes justified
- ☐ Often justified
- ☐ Always justified

21. Do you take ethical implications into account when making decisions about physical restraint?

- ☐ Always
- ☐ Often
- ☐ Sometimes
- ☐ Rarely
- ☐ Never

**22. Which ethical principles seem most important to you when deciding to use physical restraint?** *(Select all that apply)*

- ☐ Beneficence (acting in the patient's best interest)
- ☐ Non-maleficence (avoiding harm)
- ☐ Autonomy (respect for patient self-determination)
- ☐ Justice (fair allocation of care)
- ☐ Dignity
- ☐ Proportionality
- ☐ Other (please specify): ___

23. Do you take legal aspects into account when making decisions about physical restraint in critical care?

- ☐ Always
- ☐ Often
- ☐ Sometimes
- ☐ Rarely
- ☐ Never

**24. How do you take legal aspects into account in these decisions?** *(Free-text response)*

__________________________________________________________

__________________________________________________________

25. Do you inform the family of the decision to use physical restraint?

- ☐ Always
- ☐ Often
- ☐ Sometimes
- ☐ Rarely
- ☐ Never

26. If yes, how would you rate the family's acceptance of this decision?

- ☐ Very poor acceptance
- ☐ Poor acceptance
- ☐ Moderate acceptance
- ☐ Good acceptance
- ☐ Very good acceptance

27. Have you observed an impact on the family due to the patient's physical restraint?

- ☐ Yes
- ☐ No

**28. What impact do you think physical restraint can have on the patient's family?** *(Select all that apply)*

- ☐ Increased anxiety or distress
- ☐ Loss of trust in the medical team
- ☐ Feelings of guilt
- ☐ Acceptance and understanding
- ☐ Sense of safety for the patient
- ☐ Other (please specify): ___

End of questionnaire - thank you for your participation.
